# The Application of Artificial Intelligence-Assisted Colposcopy in a Tertiary Care Hospital within a Cervical Pathology Diagnostic Unit

**DOI:** 10.3390/diagnostics12010106

**Published:** 2022-01-04

**Authors:** Aleksandra Zimmer-Stelmach, Jan Zak, Agata Pawlosek, Anna Rosner-Tenerowicz, Joanna Budny-Winska, Michal Pomorski, Tomasz Fuchs, Mariusz Zimmer

**Affiliations:** Department of Obstetrics and Gynecology, Wroclaw Medical University, Borowska 213, 50556 Wroclaw, Poland; jan.zak89@gmail.com (J.Z.); agata.pawlosek@umw.edu.pl (A.P.); anna.rosner-tenerowicz@umw.edu.pl (A.R.-T.); joanna.budny-winska@umw.edu.pl (J.B.-W.); michal.pomorski@umw.edu.pl (M.P.); tomasz.fuchs@umw.edu.pl (T.F.); mariusz.zimmer@umw.edu.pl (M.Z.)

**Keywords:** colposcopy, artificial intelligence, AI, cervical pathology, new technologies, cervical cancer, cervical intraepithelial neoplasia

## Abstract

The rising global incidence of cervical cancer is estimated to have affected more than 600,000 women, and nearly 350,000 women are predicted to have died from the disease in 2020 alone. Novel advances in cancer prevention, screening, diagnosis and treatment have all but reduced the burden of cervical cancer in developed nations. Unfortunately, cervical cancer is still the number one gynecological cancer globally. A limiting factor in managing cervical cancer globally is access to healthcare systems and trained medical personnel. Any methodology or procedure that may simplify or assist cervical cancer screening is desirable. Herein, we assess the use of artificial intelligence (AI)-assisted colposcopy in a tertiary hospital cervical diagnostic pathology unit. The study group consisted of 48 women (mean age 34) who were referred to the clinic for a routine colposcopy by their gynecologist. Cervical images were taken by an EVA-Visualcheck ^TM^ colposcope and run through an AI algorithm that gave real-time binary results of the cervical images as being either normal or abnormal. The primary endpoint of the study assessed the AI algorithm’s ability to correctly identify histopathology results of CIN2+ as being abnormal. A secondary endpoint was a comparison between the AI algorithm and the clinical assessment results. Overall, we saw lower sensitivity of AI (66.7%; 12/18) compared with the clinical assessment (100%; 18/18), and histopathology results as the gold standard. The positive predictive value (PPV) was comparable between AI (42.9%; 12/28) and the clinical assessment (41.8%; 18/43). The specificity, however, was higher in the AI algorithm (46.7%; 14/30) compared to the clinical assessment (16.7%; 5/30). Comparing the congruence between the AI algorithm and histopathology results showed agreement 54.2% of the time and disagreement 45.8% of the time. A trained colposcopist was in agreement 47.9% and disagreement 52.1% of the time. Assessing these results, there is currently no added benefit of using the AI algorithm as a tool of speeding up diagnosis. However, given the steady improvements in the AI field, we believe that AI-assisted colposcopy may be of use in the future.

## 1. Introduction

The World Health Organization stated that in 2020, 604,127 women were diagnosed with cervical cancer and 341,831 women are estimated to have died from the disease worldwide [1]. The American Institute of Cancer Research lists cervical cancer as the fourth most prevalent cancer in women, and it accounts for 6.9% of total cancer incidence in the female population after breast, colorectal and lung cancer. The high incidence of cervical cancer makes it the most prevalent malignancy among all gynecological cancers [2].

Novel methods of prevention, screening, diagnosis and the identification of etiological risk factors have transformed cervical cancer into a preventable, as well as manageable, disease if diagnosed in early stages. However, this is accurate only when analyzing countries with well-established cervical cancer screening policies, including national guidelines on routine cervical cancer screening and follow-up. The ability to perform routine cervical cancer screening may vary with regards to socioeconomic status, as well as access to health services. Any method, procedure or novel technology that speeds up the screening process or decreases the burden on medical services is therefore beneficial.

Routine cervical cancer screening and follow-up programs have been developed in various countries. The effects of routine screening have been shown to prevent the incidence of localized and non-localized cervical cancers [1]. These decreases have been attributed to frequent cytological tests and colposcopy. Their role in a potential decrease in cervical cancer due to the implementation of molecular tests, Human papilloma virus (HPV) serotype identification, as well as HPV vaccines, is yet to be confirmed in large-population and long-term studies. The implementation of HPV vaccines for the prevention of cervical cancer is, however, a milestone in gynecological medicine [2].

Early cervical cancer screening was based purely on performing regular PAP smears (most commonly every 3 years) with a cytological assessment performed by a pathologist. Overall, the frequency of testing is correlated with a given epidemiological strategy, or more closely, the allocation of public resources to optimize medical economics. In essence, a poorer nation whose primary cause of premature death is related to infectious diseases will invest in sanitation rather than cervical cancer screening programs. Regardless, considering the high global incidence and mortality of cervical cancer, any novel procedure or method aimed at simplifying screening and diagnosis may be beneficial as it may decrease medical costs.

Early classification of cervical dysplasia, or more specifically, the classification of the lesions themselves, was described by the Papanicolau System. The Papanicolau system was further superseded with the Bethesda Classification. Later on, Harald zur Hausen’s discovery of HPV infection as a driver in the development of most cervical cancer cases changed the diagnostic landscape in the direction of detecting viral nucleic acids within the cells themselves. Currently, the approach towards cervical cancer screening is directed towards HPV screening and prevention [3]. The diagnostic process is expanded further by combining Liquid-Base Cytology (LBC) with simultaneous HPV genotyping combined with ‘pathological replication’ markers such as p16 and ki67. This methodology allows for early diagnosis of cervical premalignant stages (HSIL, ASC-H represented by a histological CIN2+ including CIN 2 and CIN3). The steady implementation of these tests currently forms part of a widely accepted standard-practice cervical cancer diagnostic protocol. It is worth mentioning that each implementation of a novel testing procedure was often met with skepticism, as well as a lack of early acceptance. Furthermore, the novelization and implementation of novel screening methods is only accepted by gynecologists who do not find the ‘novelty approach’ to be too complicated. This is especially true in countries where national guidelines refer only to publicly funded screening methods such as the PAP smear alone.

Since the implementation of HPV genotyping, there have been numerous advances in cervical cancer screening approaches. They are: classical conventional PAP smear (least advised by most medical boards [3,4,5]); primary HPV screening alone or with simultaneous LBC; alternatively, primary HPV screening with p16/ki67 assessment altogether; or simply HPV extended genotyping as a diagnostic starting point. In certain cases, even the presence of high-risk HPV nucleic acid is an indication to perform a colposcopy. Considering everything, there is, however, no unified recommended worldwide screening system that is optimal for everyone. The reason may be due to lack of access to medical services as well as accredited molecular laboratories that are required to perform the necessary tests. In essence, the main limiting factor for why patients do not receive the best standard of care is due to limited healthcare funding. This is apparent in countries that offer privately funded healthcare systems such as the United States, where primary HPV testing is recommended to be performed every 5 years as the best approach [5]. Contrary to Western countries, health systems with publicly funded healthcare systems often do not reimburse the extra cost of LBC and/or HPV testing, not to mention p16/ki67 staining. In the United Kingdom (UK), the National Health Service (NHS) made a strong effort to test patients using primary HPV identification and genotyping, regardless of funding. With regards to treatment, it is accepted that a paradigm shift regarding the transition towards primary HPV testing has been made. In the case of the United Kingdom’s National Healthcare System, the drive to increase HPV testing failed because the histopathological and molecular biology laboratories were unable to handle the sudden increase in patient samples; or in other words, consumer demand [4]. As mentioned by Hashim et al. [6] it is crucial to improve the treatment of HPV positive patients with positive cytology by differentiating the HPV genotype and choosing more conservative management for women with ASC-US/LSIL caused by other high-risk HPV than HPV16/18.

What most cervical cancer screening recommendations agree on is the role of colposcopy as a key diagnostic tool. After a pathological finding is discovered in the first stage, colposcopy is the second stage of the diagnostic process. Performing a colposcopy allows the doctor to take biopsies, and this procedure therefore allows for a definitive answer to the histopathological nature of the tissue being acquired. Professional colposcopy is performed in an expert referral center, guaranteeing optimal colposcopy protocol selection, accompanied by an adequate and representative biopsy from the cervix and, if necessary, a cervical canal curettage [3,5,7,8,9]. In cases where it is not possible to perform a colposcopy, the WHO recommends proceeding with treatment without obtaining a biopsy [3]. As was the case with the NHS, creating appropriate healthcare and molecular laboratory infrastructure can become difficult and limit the feasibility of a screening campaign. An insufficient number of expert centers or lack of medical resources may be a limiting factor in treating an overburdening influx of patients. In these situations where expert opinion is not available on-site, additional tools might be helpful. One of such tools, gaining more and more acceptance, is Artificial Intelligence (AI)-assisted colposcopy [10,11] implemented at the screening level.

The idea of AI in medicine is not a new one. The concept of emulating the human mind using computer algorithms to distinguish between normal and abnormal findings has been tested in multiple scientific fields, including medicine. Common applications of AI in medicine have been reported in gastrointestinal endoscopy, eye fundus examination in ophthalmology, radiology and even (but not limited to) dermatology [12]. Colposcopy itself, as a test, relies strongly on the impression of the performing specialist based on acquired images of the cervix. The ability of a scientist to develop a keen eye that detects pathologies takes many hundreds of patients as well as clinical training. In the case of AI-assisted colposcopy, saved digital images of a cervix are perfect candidates as they may be accumulated and later assessed using an AI algorithm. Furthermore, the ability of an AI algorithm to classify images as normal versus abnormal is dependent on the size and quality of the database used, as well as feedback whether the algorithm is correct or not. Implementing AI to assess digital images of the cervix is a perfect field to assess the feasibility of AI-based technology. Some potential caveats of implementing AI in colposcopy which seem to be at least partially resolved were: (1) the three-dimensional nature of the real-time assessment of the cervix made by an expert actually taking the photos—the archived colpograms were saved as a simple still frame leading to a potential loss of the 3D effect, (2) a disturbed, difficult-to-assess image after multiple staining applications with both acidic acid and iodine solution (Lugol’s solution). Considering the first caveat regarding the 3D nature of the cervix, the issue was resolved by the hardware of the portable colposcope by reducing the aperture of the lens. This was supplemented with a high enough resolution of the digital colposcope and an appropriate algorithm ‘distinguishing’ the depth of the picture taken [13]. Regarding the second caveat, it was empirically decided that acetic acid was best suited for AI colposcopic analysis. More precisely, careful analysis was performed regarding the necessity of double staining based on the sensitivity of acidic acid interference with proteins in the cervical epithelium versus iodine Lugol’s solution avidity with the glycogen in the intermediate layer of the squamous epithelium of the cervix. As mentioned previously, most AI-assisted colposcopes settle for acetic acid staining, claiming it is overall clearer to assess. The necessity to perform full colposcopy with or without the Schiller test (aqueous solution of Iodine) remains controversial in different medical societies; for example, the Spanish AEPCC supports the Schiller test as part of its guidelines [14]. Others, such as the Polish Society of Obstetricians and Gynaecologists and the Polish Society of Colposcopy and Cervical Pathophysiology, do not recommend the Schiller test as routine practice [7]. It is worth mentioning that the 2011 International Federation of Cervical Pathology and Colposcopy guidelines and Colposcopic Classification mentioned Iodine staining among ‘non-specific’ abnormal colposcopic findings [15].

The gold standard for the assessment of cervical lesions, as well as any cancer diagnostic process, has always been the histopathological analysis of biopsy results. The success of this gold standard procedure is dependent on the proper determination of the actual places where a targeted cervical biopsy is performed. Multiple classifications of the patterns appearing on the cervix after various staining procedures have been described (Hinselmann [14,16], Coppleson [14,17], Reid [18], IFCP [19], SWEDESCORE [20]). Proper diagnosis and naming criteria, as well as adequate classification, rely on the expertise of the medical professional performing a colposcopy and therefore are very subjective. In the absence of the clearly abnormal structures within the transformation zone, for most cases, a random biopsy is advised [7]. It must be acknowledged that biopsy results may take several weeks and thus may delay the implementation of adequate treatment. The results of the biopsy may imply further treatment and/or diagnostics.

Ideally, AI-assisted colposcopy may decrease some burden of interpreting cervical patterns after acetic acid staining, and after a short computer analysis may give a binary ‘yes/no’ answer to whether the examined cervix looks suspicious (biopsy strongly advised) or not. This approach may assist beginner-level medical professionals to learn, as well as give the patient peace of mind while waiting for biopsy results. What is apparent is that the implementation of AI-assisted colposcopy is dependent on acquiring a digital image of the cervix, and hence may be performed by beginner-level medical professionals. There is ample utility in AI-assisted colposcopy in teaching and assessing the clinical impression of even the most advanced medical professionals. This approach once again allows a doctor to be more confident when treating their patients, and improves patient confidence. In an ideal future sense, AI could point out the suspicious areas within the transformation zone of the cervical epithelium, making the biopsy even simpler. 

The algorithms applied in the AI colposcopy devices available on the market vary and are often patent-protected. The main idea behind them is the encrypted cloud-based storage space where all colpograms taken in the medical facility are being sent and stored [10,21,22]. The analysis is based on layers of analysis that allow very complex images to be taken apart, and after passing multiple layers may give a binary “yes” or “no” answer.

More precisely, after splitting the image into 3D-like tiny pixels, each of the ‘regions’ of the cervix are classified as normal or abnormal in comparison to thousands of pictures previously analyzed by both: human expert and AI-machine [22]. That is how an AI algorithm may be able to determine a normal versus abnormal image; however, this is different from a simple atlas-like comparison of the normal/abnormal pattern. The algorithm not only processes the information stored in a pixel, but also analyzes its location within the cervix. Based on that assessment, an algorithm is able to determine patterns that may give an indication as to how ‘risky’ this exact spot might be, based on previously analyzed lesions. After summing the normal/abnormal regions and taking into consideration the ‘importance’ of each spot with regards to cervical abnormality (e.g., transformation zone versus beyond transformation zone) the final assessment is made using another layer of more specific algorithm. The final result states that a given cervical image is normal or abnormal and leaves the doctor with the decision to make regarding further tests or biopsies to perform.

Herein we define the term “artificial intelligence (AI) algorithm” as it is used in the study. There are many branches of AI, such as facial and pattern recognition, machine learning, neural networks and deep neural networks. Briefly, the EVA Visualcheck^TM^ algorithm, which we have used in the study, is a convolutional neural network clinical decision tool used to correctly identify ≥CIN2+ cervical pathologies (schematic presentation in the Supplementary Materials).

Convolutional neural networks are composed of visible and hidden layers that are themselves composed of nodes. It is important to note that the number of layers and nodes may change. Furthermore, the weights and biases that link one layer to the next may also be different and change. For this reason, you could have two convolutional neural networks that have the same architecture but give different results, as they may have different weights and biases between the nodes and layers. This is why many refer to a convolutional neural network as a black box (Supplementary Materials). We use the term “black-box classifier”, because the classifier parameters will change with regards to a teaching database. The teaching database primes the black box classifier to correctly identify ≥CIN2+ vs. <CIN2+ cervical images. Each layer is interconnected to the next layer with different weights and biases. As mentioned previously, weights and biases are primed using a teaching database that provides feedback to the neural network because the output results are known [23].

In our study, a given cervical image is cropped and resized for the first visual input layer of the convolutional neural network. The AI algorithm takes a given cervical image as the input, runs it through the previously described black box classifier, and returns a binary answer of either a ‘normal’ (<CIN2+) or ‘abnormal’ (≥CIN2+).

## 2. Materials and Methods

The study was a retrospective analysis of 48 colposcopy examinations performed at the 2nd Department of Obstetrics and Gynaecology, Wroclaw Medical University, Poland, in the Cervical Pathology Diagnostic Unit.

All patients were referred to the Cervical Pathology Diagnostic Unit due to either an abnormal overall clinical impression of the cervix in a speculum examination, abnormal PAP smear (ASCUS+) and/or abnormal PAP smear with a positive High-risk (HR) HPV test. A total of 51 patients were assessed; however, only 48 were chosen for the study. 

A colposcopy with acetic acid applied for 1 min to the cervix and a cervical biopsy—either targeted, or in the absence of a pathological pattern, a randomized biopsy—was performed in all subjects. Additionally, all patients had their cervix assessed as normal/abnormal by a board-certified gynecologist with expertise and training in cervical pathology. The acquired images/colpograms were assessed using an artificial intelligence-based algorithm called Visualcheck ^TM^. The algorithm used is a built-in feature of the colposcope-like instrument registered within the European Union under the brand name EVA System [24,25].

The one-sided blind study was performed such that there was no knowledge of the Visualcheck^TM^ algorithm ruling until all subjects were tested, assessed by the gynecologist and until all of the histopathological results were returned by a pathologist. The AI-based colposcope qualification of the cervix as a normal or abnormal was not known to the examiner. 

Out of 51 initial patients, there was one non-diagnostic biopsy taken in one case, and this case was excluded from the study. In two cases, due to a type 3 transformation zone, a full visual assessment was impossible, and 48 patients were therefore included in the statistical analysis. Altogether, 48 cases with complete data regarding recent PAP smear result, colposcopy-expert assessment, AI-based colposcope assessment and histopathological biopsy sample results were included in the study.

## 3. Results

48 patients with abnormal PAP smear, and/or positive HR-HPV, and/or abnormal subjective morphology of the cervix were qualified for colposcopy based on referrals from their gynecologist. The mean age of the patient was 34 years old (Min: 20, Max: 59, SD 9.29). Among 48 PAP smear results, the most common reason for the referral was low-grade squamous intraepithelial lesion (LSIL) (17 cases, 35.4%), followed by high-grade squamous intraepithelial lesion (HSIL) (10 cases, 20.8%). The detailed results are presented in Table 1.

All of the patients who qualified for the study underwent a standard colposcopy with acetic acid staining (for 1-min) and subsequently a targeted or randomized biopsy. A minimum of 4 samples were taken from each cervix. A colposcopy was first performed using a conventional digital colposcope and then, after staining, an additional image with a digital AI colposcope was taken. The examiner was not aware of the mobile AI-colposcope (EVA System, Visualcheck^TM^) ruling until all patients were examined and all histopathological samples were assessed by a pathologist. The study therefore meets the criteria of a blind trial.

The AI-based algorithm assessed 28 out of 48 cervical images as abnormal (58.3%; Table 2). This was smaller than the colposcopist’s subjective opinion, which marked 43 out of 48 as abnormal (89.6%; Table 3). There was a statistically significant difference in the assessments between the AI algorithm and an experienced colposcopist (*p* = 0.003). The comparison of the AI-based mobile colposcope assessment with the gold standard, a biopsy result, showed the results came close to being statistically significant if we considered the abnormality cut-off point at the level of CIN2+ (*p* = 0.062); The agreement of AI-colposcopy and histopathological results was only 54.2% (normal vs. abnormal defined as a CIN2+).

The discrepancy between an expert analysis with biopsy results if we set the pathology mark at the level of CIN2+ is significant (*p* < 0.001). Out of 43 patients assessed as abnormal by the clinician, 18 turned out to have a true pathological finding of CIN2+. The agreement between a clinician’s opinion and histopathology results (normal vs. abnormal defined as ≥CIN2+) was found in 47.9% of the cases.

Regarding the agreement between the AI algorithm result and a clinical assessment of either a normal or abnormal cervix, it was congruent for 60% of the cases.

Based on the above, the PPV, based on the histopathological biopsy as a gold standard, for the AI algorithm detection of CIN2+ was 42.9%, NPV 70%, sensitivity 66.7% and specificity 46.7%. The PPV of the colposcopist in our study was 41.8%, NPV 100%, sensitivity 100%, specificity 16.7%.

The results overall may suggest that experienced clinicians may overdiagnose cervical pathologies. The obvious beneficial rationale is to reduce false-negative results. Therefore, patients referred to the tertiary medical center risk overdiagnosis of the pathological appearance of the cervix when a colposcopy is performed, even by an experienced clinician. The reduction of false-negative results come at the cost of low specificity of 16.7%. On the other hand, AI-assisted colposcopy was able to detect pathologies similar to that of a trained physician PPV (42.9% vs. 41.8%), with visibly lower sensitivity (66.7% vs. 100%) but simultaneously an increased specificity of 46.7% (vs. 16.7%).

A clear limitation of the study was the low number of patients enrolled. We are looking forward to expanding the study group in future studies in order to increase the value of calculated PPV, NPV, sensitivity and specificity with CIN2+ as a cut-off point. CIN2+ is currently considered a true precancerous stage in the pathogenesis of HPV-related cervical cancer.

## 4. Discussion

AI is a useful tool that has the ability to emulate and expand human diagnostic ability. In the last few years, the application of AI in medicine has become a hot topic in modern science and technology. It is believed that in the future, AI may improve the efficiency of diagnosis, reduce the workload of clinicians and even improve the effect of treatment and prognosis. Furthermore, AI has been shown to be more effective at recognizing certain patterns than the human brain is. Radiology is the branch that has been the most upfront and welcoming to the use of novel technology such as AI. In 2020, Baldwin et al. conducted a validation study by retrospectively collecting the database of pulmonary nodules of size 5–15 mm which were noted incidentally from three hospitals in the United Kingdom. In this study, an AI algorithm, the lung cancer prediction convolutional neural network (LCP-CNN), was compared with the Brock University model, as advised in UK guidelines. The LCP-CNN was found to outperform the Brock model, which itself represents the most discriminative baseline risk model available [26]. 

In gynecology, the cervical cancer diagnostic protocol which, in the presence of HR-HPV and/or abnormal PAP smear, requires a colposcopy, and therefore collecting the diagnostic tissue samples before introducing any potentially invasive treatment, seems to be the perfect ground for the development of AI-assisted tools.

A few large studies in the field of AI in cervical diagnosis have been published. The interest, despite boosting colposcopy, is also directed towards supporting cytology and the assessment of cervical epithelium at the level of the pathology laboratory [10,21]. 

A significant study by Cho et al. concluded that deep-learning AI algorithms may support underexperienced clinicians in the decision of whether to perform a cervical biopsy or not [27]. Miyagi et al. in their study established that the classifier using deep-learning technology is better than that of oncologists, although the results were not statistically significant [28]. They pointed out that with proper training and technological progress there might be a spot for AI devices in the process of cervical cancer screening. Promising results have been reported in a Chinese large-scale study (over 19,000 patients) by Xue et al. Researchers found the agreement between Colposcopic Artificial Intelligence Auxiliary Diagnostic System (CAIADS)-graded colposcopic impressions and pathology findings was higher than that of colposcopies interpreted by colposcopists (82.2% versus 65.9%) [22]. A very interesting branch of the quoted study was the system’s ability to point out biopsy sites based on the suspicious pattern (recognized by the CAIADS) and how accurately it was suggested. The mobile colposcope we tested in our study, at least so far, is lacking this functionality.

The limitation of our study is definitely in the numbers of the patients enrolled. Unfortunately, the possibility of testing the mobile AI-induced colposcope coincided in time with the global COVID-19 pandemic, resulting in severely reduced patient referrals and patient compliance with regards to showing up to planned procedures. This was all despite the huge efforts from both the Polish Society of Obstetricians and Gynecologists and the Polish Society of Colposcopy and Cervical Pathophysiology, which issued joint special guidelines for the COVID-pandemic [8], encouraging patients, especially those with high-grade cervical pathologies at the screening level, to proceed with the colposcopy and, if necessary, to obtain a biopsy to enable further treatment.

## 5. Conclusions

AI-assisted colposcopy seems, in theory, to be a good addition in a cervical diagnostic unit that performs colposcopy. Given the inadequate results, AI-assisted colposcopy cannot replace a qualified colposcopist. Currently, it is an additional tool, possibly a tool that is reassuring to the patient at the screening level, in a private-practice setting, in cases where there are no immediate pathological findings nor risk factors present. We wish to stress that there is no argument being made that AI-assisted colposcopy is able at the present time to replace well-established screening tests (PAP smear and/or HPV primary testing). On the other end of the spectrum, the AI-assisted colposcope may be a ‘patient-motivating’ pathology detector if there are obvious, high-grade abnormalities that are visualized, and this may improve patient compliance in seeking treatment. The quality of digital images and easy ergonomic interface are all pleasant to work with. However, they do not replace a comprehensive assessment of the patient. This can only be performed by an experienced gynecologist who is able to assess the whole clinical picture. These additional mediating factors may be: HPV status, previous screening results, age, etc. For an algorithm to accommodate all those additional factors, there would need to be a much larger study population. While our results show that there is no added benefit of using AI-assisted colposcopy, we do believe there definitely is a future for this technology in the clinic. In the end, an experienced gynecologist must make and take responsibility for the final determination and assess whether what the AI-algorithm is saying makes sense. A future, possibly involving the potential of AI-induced colposcope into a cervical biopsy targeting, seems a consecutive next step in the AI-based colposcopy development we are looking for.

## Figures and Tables

**Table 1:** PAP smear results among the study group.

PAP Smear	No	%	CIN2+	% CIN2+ among Selected PAP	% CIN2+ among All CIN2+
Normal (NILM)	6	12.5%	0	0%	0%
Low-Grade (ASCUS, LSIL)	21	43.8%	3	3/21 14.3%	3/18 16.7%
High Grade (AGC, ASC-H, HSIL)	20	41.7%	15	15/20 75%	15/18 83.3%
indeterminate	1	2%	0	0%	0%
Total	48	100%			100%

**Table 2:** Histopathological assessment of the biopsy samples compared to the AI-induced colposcopy assessment.

	*N* = 48				
	Histopathology Result (CIN2+/Normal)		AI-Colposcope Assessment (Normal/Abnormal)		
	No	%	No		%
Normal	30	62.5%	20		41.7%
Abnormal	18	37.5%	28		58.3%
AI-induced colposcope vs. histopathology	PPV		42.9%	NPV	70%
	sensitivity		66.7%	specificity	46.7%

**Table 3:** Histopathological assessment of the biopsy samples compared to the expert colposcopist assessment.

	*N* = 48				
	Histopathology Result (CIN2+/Normal)		Colposcopist Assessment		
	No	%	No	%	
Normal	30	62.5%	5	10.4%	
Abnormal	18	37.5%	43	89.6%	
colposcopist assessment vs. histopathology	PPV		41.8%	NPV	100%
	sensitivity		100%	specificity	16.7%

## Data Availability

Not applicable.

## Supplementary Materials

The following are available online at https://www.mdpi.com/article/10.3390/diagnostics12010106/s1, Figure S1: Schematic study workflow with AI assisted colposcopy.

## References

[1] GLOBOCAN (2020). Cervix uteri Source: Globocan 2020. Int. Agent Res. Cervix. Uteri..

[2] Ferlay J., Colombet M., Soerjomataram I., Mathers C., Parkin D.M., Piñeros M., Znaor A., Bray F. (2019). Estimating the global cancer incidence and mortality in 2018: GLOBOCAN sources and methods. Int. J. Cancer.

[3] WHO (2013). Guidelines for Screening and Treatment of Precancerous Lesions for Cervical Cancer Prevention. WHO Guidel [Internet]. http://www.who.int/reproductivehealth/publications/cancers/screening_and_treatment_of_precancerous_lesions/en/index.html.

[4] Regan L., Music R., Martin-Hirsch P., Kasliwal A. (2019). RCOG, BSCCP, FSRH and Jo’ s Cervical Cancer Trust Joint Position State-ment. Cerv. Cancer Screen..

[5] Fontham E.T.H., Wolf A.M.D., Church T.R., Etzioni R., Flowers C.R., Herzig A., Guerra C.E., Oeffinger K.C., Shih Y.T., Walter L.C. (2020). Cervical cancer screening for individuals at average risk: 2020 guideline update from the American Cancer Society. CA Cancer J. Clin..

[6] Hashim D., Engesæter B., Skare G.B., Castle P.E., Bjørge T., Tropé A., Nygård M. (2020). Real-world data on cervical cancer risk stratification by cytology and HPV genotype to inform the management of HPV-positive women in routine cervical screening. Br. J. Cancer.

[7] Jach R., Mazurec M., Trzeszcz M., Bartosinska-Dyc A., Galarowicz B., Kedzia W., Pitynski K. (2020). COLPOSCOPY 2020-COLPOSCOPY protocols a summary of the clinical experts consensus guidelines of the Polish society of colposcopy and cervical pathophysiology and the Polish society of gynecologists and obstetricians. Ginekol. Pol..

[8] Jach R., Mazurec M., Trzeszcz M., Zimmer M., Kedzia W., Wolski H. (2021). Cervical cancer screening in Poland in current SARS-CoV-2 pandemic: Interim guidelines of the Polish Society of Gynecologists and Obstetricians and the Polish Society of Colposcopy and Cervical Pathophysiology—A summary January 2021. Ginekol. Pol..

[9] Perkins R.B., Guido R.S., Castle P.E., Chelmow D., Einstein M.H., Garcia F., Schiffman M. (2020). 2019 ASCCP Risk-Based Management Consensus Guidelines for Abnormal Cervical Cancer Screening Tests and Cancer Precursors. J. Low Genit. Tract. Dis..

[10] Bao H., Sun X., Zhang Y., Pang B., Li H., Zhou L., Wang L. (2020). The artificial intelligence-assisted cytology diagnostic system in large-scale cervical cancer screening: A population-based cohort study of 0.7 million women. Cancer Med..

[11] Bedell S.L., Goldstein L.S., Goldstein A.R., Goldstein A.T. (2020). Cervical Cancer Screening: Past, Present, and Future. Sex. Med. Rev..

[12] Xue P., Qiao Y.L., Jiang Y. (2018). Application of artificial intelligence in diagnosis of medical endoscope. Zhonghua Zhong Liu Za Zhi Chin. J. Oncol..

[13] Xue P., Ng M.T.A., Qiao Y. (2020). The challenges of colposcopy for cervical cancer screening in LMICs and solutions by artificial intel-ligence. BMC Med..

[14] Torné A., del Pino M., Andía D., Castro M., de la Fuente J., Hernández J. (2018). Colposcopy guidelines. Stand. Qual..

[15] Bornstein J., Bentley J., Bösze P., Girardi F., Haefner H., Menton M., Perrotta M., Prendiville W., Russell P., Sideri M. (2012). 2011 Colposcopic Terminology of the International Federation for Cervical Pathology and Colposcopy. Obstet. Gynecol..

[16] Sellors J.W., Sankaranarayanan R. (2013). Colposcopy and Treatment of Cervical Intraepithelial Neoplasia. A Beginner’s Manual.

[17] Coppleson M., Dalrymple C., Atkinson K.H. (1993). Colposcopic differentiation of abnormalities arising in the transformation zone. Obstet. Gynecol. Clin. N. Am..

[18] Reid R., Scalzi P. (1985). Genital warts and cervical cancer. VII. An improved colposcopic index for differentiating benign papilloma viral infections from high-grade cervical intraepithelial neoplasia. Am. J. Obstet. Gynecol..

[19] Stafl A. (1976). New nomenclature for colposcopy. Report of the committee on terminology. Obstet. Gynecol..

[20] Bowring J., Strander B., Young M., Evans H., Walker P. (2010). The Swede Score: Evaluation of a scoring system designed to improve the predictive value of colposcopy. J. Low. Genit. Tract Dis..

[21] Wentzensen N., Lahrmann B., Clarke M.A., Kinney W., Tokugawa D., Poitras N., Locke A., Bartels L., Krauthoff A., Walker J. (2020). Accuracy and Efficiency of Deep-Learning–Based Automation of Dual Stain Cytology in Cervical Cancer Screening. J. Natl. Cancer Inst..

[22] Xue P., Tang C., Li Q., Li Y., Shen Y., Zhao Y., Chen J., Wu J., Li L., Wang W. (2020). Development and validation of an artificial intelligence system for grading colposcopic impressions and guiding biopsies. BMC Med..

[23] Fernando’s K.C., Freitas T., Zall Y., Nissim R., Levitz D. Device Impact on Machine Learning Classifier Accuracy in Detecting Cervical Dysplasia. Mobile ODT–Oral Presentation: 2020 ASCCP Annual Scientific Sessions. https://www.mobileodt.com/medical-research/device-impact-on-machine-learning-classifier-accuracy-in-detecting-cervical-dysplasia/.

[24] Mink J., Peterson C. (2016). MobileODT: A case study of a novel approach to an mHealth-based model of sustainable impact. mHealth.

[25] Goldstein A., Lei Y., Goldstein L., Goldstein A., Bai Q.X., Felix J., Lipson R., Demarco M., Schiffman M., Egemen D. (2020). A rapid, high-volume cervical screening project using self-sampling and isothermal PCR HPV testing. Infect. Agents Cancer.

[26] Baldwin D.R., Gustafson J., Pickup L., Arteta C., Novotny P., Declerck J., Gleeson F.V. External Validation of a Convolutional Neural Network Artificial Intelligence Tool to Predict Malignancy in Pulmonary Nodules Lung Cancer. http://thorax.bmj.com/.

[27] Cho B.-J., Choi Y.J., Lee M.-J., Kim J.H., Son G.-H., Park S.-H., Kim H.-B., Joo Y.-J., Cho H.-Y., Kyung M.S. (2020). Classification of cervical neoplasms on colposcopic photography using deep learning. Sci. Rep..

[28] Miyagi Y., Takehara K., Miyake T. (2019). Application of deep learning to the classification of uterine cervical squamous epithelial lesion from colposcopy images. Mol. Clin. Oncol..

