# Supplementary figures and images for: The Application of Artificial Intelligence-Assisted Colposcopy in a Tertiary Care Hospital within a Cervical Pathology Diagnostic Unit
Source: Diagnostics (Basel). 2022 Jan 4;12(1):106. doi: 10.3390/diagnostics12010106 (PMC8774766; doi:10.3390/diagnostics12010106)

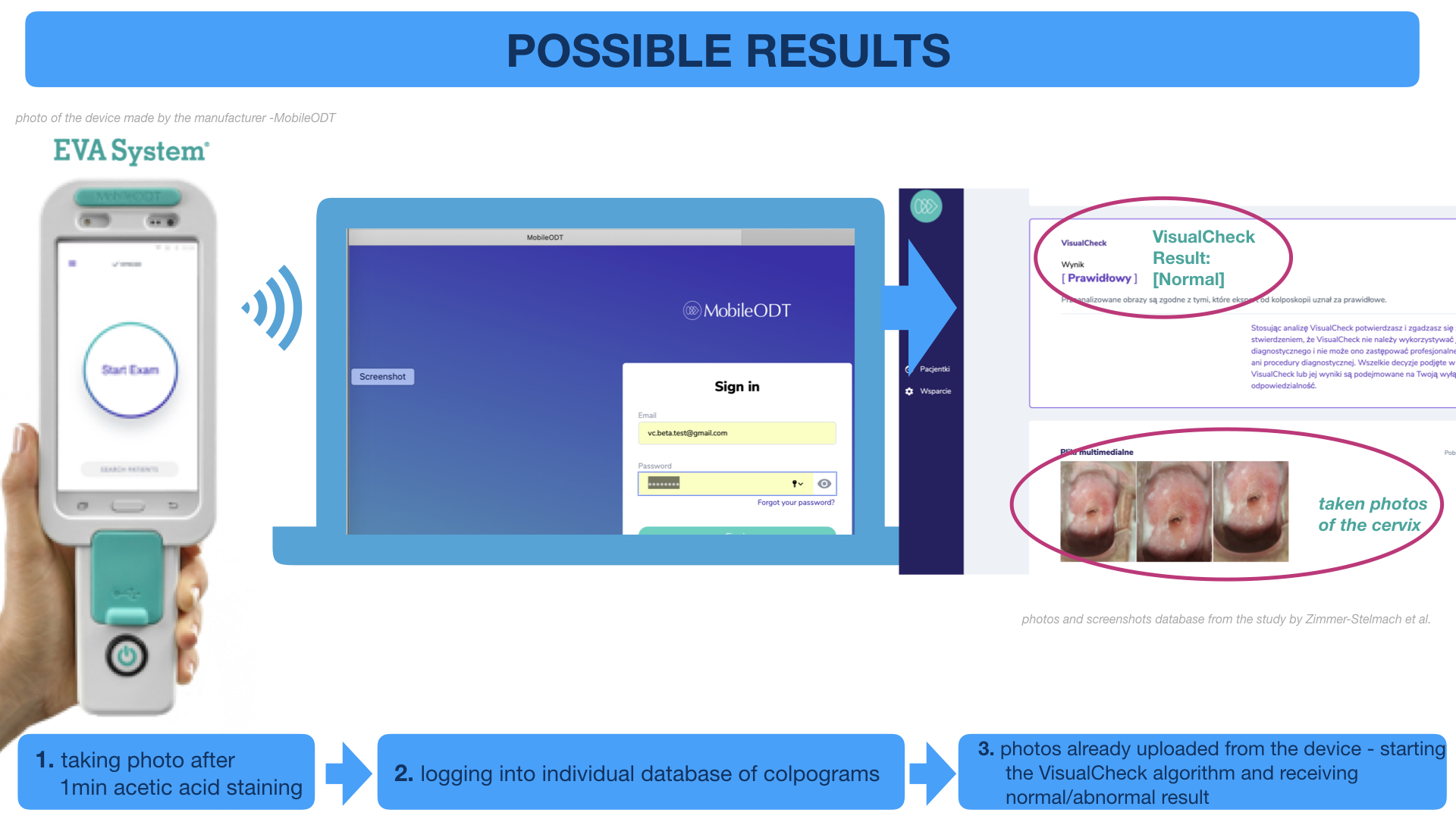

Supplement: Supplementary file 1 [file diagnostics-12-00106-s001.zip › Figure S1-002.jpeg]

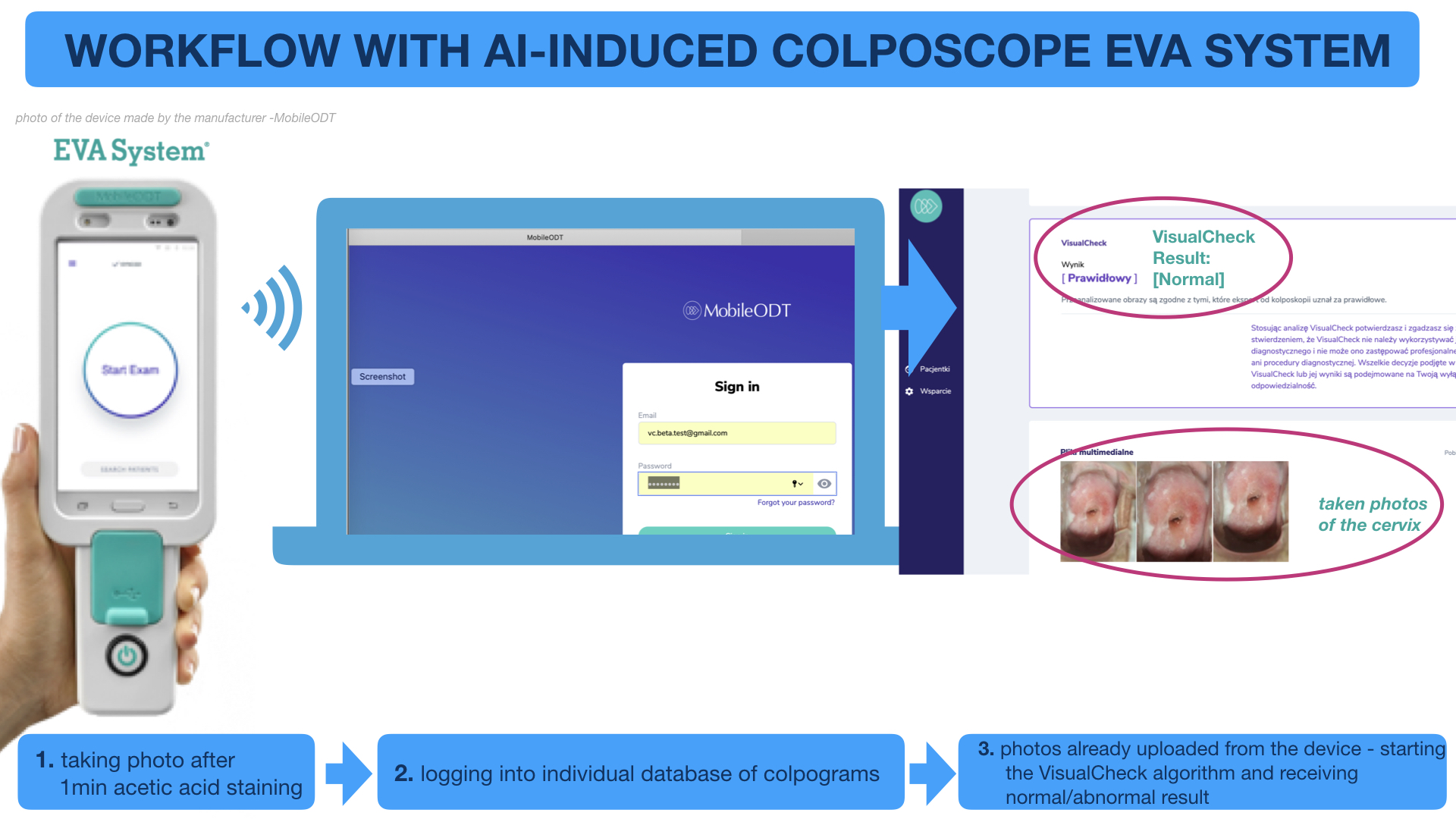

Supplement: Supplementary file 1 [file diagnostics-12-00106-s001.zip › Figure S1-001.jpeg]

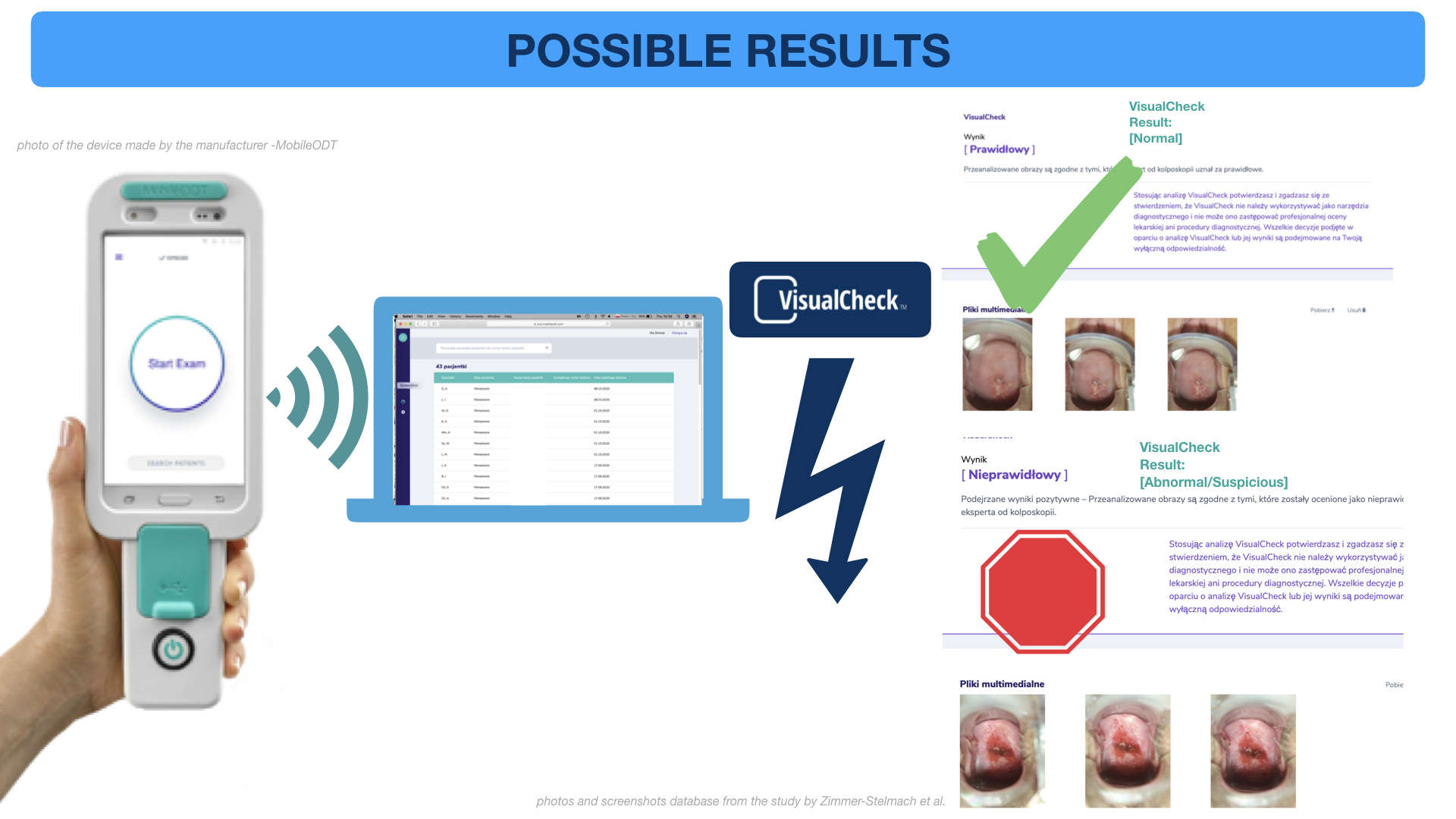

Supplement: Supplementary file 1 [file diagnostics-12-00106-s001.zip › Figure S1-003.jpeg]

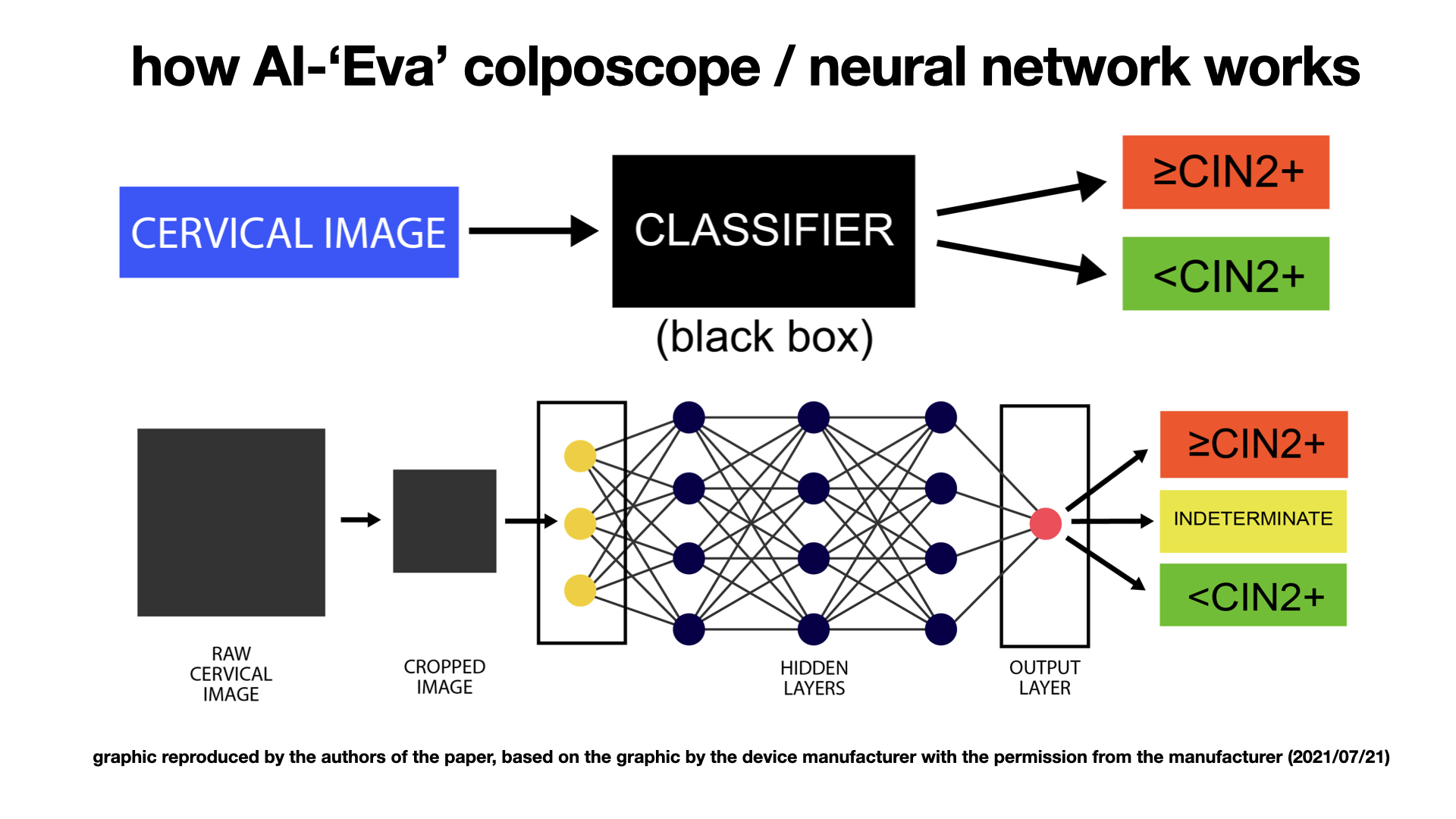

Supplement: Supplementary file 1 [file diagnostics-12-00106-s001.zip › Figure S1-004.jpeg]
